# Neurological manifestations of lysosomal storage diseases

**DOI:** 10.1097/MS9.0000000000002611

**Published:** 2024-09-30

**Authors:** Chukwuka Elendu, Emmanuel A. Babawale, Festus O. Babarinde, Olusola D. Babatunde, Christopher Chukwu, Sobechukwu F. Chiegboka, Omotola P. Shode, Jide K. Ngozi-ibeh, Anthonia Njoku, Mary N. Ikokwu, Grace U. Kaka, Jemilah I. Hassan, Oluwasunmisola O. Fatungase, Tolulope Osifodunrin, Chidi A. Udoeze, Victor I. Ikeji

**Affiliations:** a Federal University Teaching Hospital, Owerri, Nigeria; b Griffith College Dublin, Dublin, Ireland; c University of Ibadan, Ibadan, Oyo, Nigeria; d Cagayan State University, Tuguegarao, Philippines; e University of Jos, Plateau State, Nigeria; f Vinnytsia National Medical University, Vinnytsia, Ukraine; g Saint James School of Medicine, Park, park Ridge, Illinois, USA; h I. Horbachevsky Ternopil National Medical University, Ternopil, Ukraine; i Imo State University, Owerri, Imo State, Nigeria; j Abia State University, Uturu, Nigeria; k University of Nigeria, Nsukka, Enugu State, Nigeria; l Nahda University, Beni Suef, Egypt; m Caucasus International University, Tbilisi, Georgia; n Ternopil National Medical University, Ternopil, Ukraine

**Keywords:** CNS involvement, lysosomal storage diseases, multidisciplinary care, neurological manifestations, treatment strategies

## Abstract

Lysosomal storage diseases (LSDs) encompass a group of rare inherited metabolic disorders characterized by the accumulation of undegraded substrates within lysosomes, leading to multisystemic manifestations, including profound neurological involvement. This article provides a concise overview of the neurological manifestations of LSDs, with a focus on central nervous system (CNS) involvement and treatment strategies. While the paper intricacies of each LSD subtype and its associated CNS manifestations, it aims to provide a summary of the essential findings and implications. The neurological manifestations of LSDs encompass a spectrum of symptoms, including cognitive impairment, motor dysfunction, seizures, and sensory deficits, which significantly impact patients’ quality of life and pose therapeutic challenges. Current treatment strategies primarily aim to alleviate symptoms and slow disease progression, with limited success in reversing established neurological damage. Enzyme replacement therapy, substrate reduction therapy, and emerging gene therapies hold promise for addressing CNS involvement in LSDs. However, challenges such as blood-brain barrier penetration and long-term efficacy remain. In addition to discussing treatment modalities, this article highlights the importance of early diagnosis, multidisciplinary care, and patient advocacy in optimizing outcomes for individuals affected by LSDs. Ethical considerations are also addressed, including equitable access to emerging treatments and integrating personalized medicine approaches. Overall, this article underscores the complex interplay between genetics, neuroscience, and clinical care in understanding and managing the neurological manifestations of LSDs while emphasizing the need for continued research and collaboration to advance therapeutic interventions and improve patient outcomes.

## Introduction

Highlights

- *CNS involvement in lysosomal storage diseases (LSDs)*: Lysosomal storage diseases frequently involve the central nervous system (CNS), leading to a variety of neurological symptoms such as cognitive decline, motor dysfunction, and seizures. These manifestations are due to the accumulation of undigested substrates in neurons and glial cells, which disrupt normal cellular function.
- *Pathophysiology of neurological damage*: The pathophysiological mechanisms underlying CNS involvement in LSDs include impaired lysosomal function, neuroinflammation, and neuronal apoptosis. These processes result in progressive neuronal damage and loss, contributing to the severity of neurological symptoms.
- *Diagnostic challenges and approaches*: Diagnosing CNS involvement in LSDs can be challenging due to the heterogeneity of symptoms and overlap with other neurodegenerative disorders. Advanced neuroimaging techniques, biochemical assays, and genetic testing are crucial for accurate diagnosis and differentiation of specific LSD subtypes.
- *Current treatment strategies*: Treatment options for CNS involvement in LSDs are limited but evolving. Enzyme replacement therapy (ERT) and substrate reduction therapy (SRT) have shown some efficacy in managing systemic symptoms but face challenges in crossing the blood-brain barrier (BBB). Emerging strategies include gene therapy, small-molecule chaperones, and BBB-penetrant therapeutics designed to directly target CNS pathology.
- *Future directions in research and therapy*: Ongoing research aims to develop more effective treatments for CNS involvement in LSDs. Innovations such as advanced gene editing techniques, novel drug delivery systems to enhance BBB penetration, and personalized medicine approaches hold promise for improving outcomes for patients with neurological manifestations of LSDs.

Lysosomal storage diseases (LSDs) are a group of rare inherited metabolic disorders caused by defects in lysosomal enzymes or transport proteins, leading to the accumulation of undegraded substrates within lysosomes. This accumulation disrupts cellular homeostasis, causing tissue damage and multisystemic manifestations, including significant neurological involvement^1–3^. The pathophysiology of LSDs varies depending on the specific enzyme deficiency and accumulated substrate. Still, a common outcome is the progressive impairment of lysosomal function, resulting in lysosomal enlargement, impaired autophagy, and cell dysfunction^4^. Neurological manifestations are a hallmark of many LSDs and can present as cognitive impairment, motor dysfunction, seizures, sensory deficits, and psychiatric symptoms^5^. The severity and progression of these symptoms vary among different LSDs and affected individuals, complicating diagnosis and management^6^. Notably, LSDs such as Tay–Sachs disease, Niemann–Pick disease type C, and Gaucher disease exhibit pronounced neurological symptoms. Tay–Sachs disease, caused by a deficiency of hexosaminidase A, leads to severe neurodegeneration and early death^7,8^. Niemann–Pick disease type C involves the accumulation of cholesterol and sphingolipids, causing progressive neurodegeneration and diverse neurological symptoms^9^. Gaucher disease, known for its hematological and skeletal effects, can also present with progressive neurological symptoms in its neuronopathic form^10,11^. Diagnosis of LSDs combines clinical evaluation, biochemical testing, and genetic analysis, with neurological symptoms often guiding initial investigations^12,13^. Current management strategies include enzyme replacement therapy (ERT) and substrate reduction therapy (SRT), though these mainly aim to alleviate symptoms and slow progression rather than reverse neurological damage^14,15^. Emerging therapies, such as gene and chaperone therapies, hold promise for addressing the underlying defects^16^. Despite advancements, challenges remain in managing neurological symptoms due to the complexity of disease mechanisms and the variable response to treatments^17,18^.

Further research is essential to understand CNS involvement better and develop targeted therapies for effective management of neurological manifestations in LSDs. This review aims to elucidate the pathophysiology of lysosomal storage diseases (LSDs) and the mechanisms underlying central nervous system (CNS) involvement. It will explore the spectrum of neurological manifestations in LSDs, including cognitive impairment, motor dysfunction, seizures, sensory deficits, and psychiatric symptoms, and discuss their impact on patients’ quality of life. The review will also evaluate diagnostic approaches and current treatment strategies, including enzyme replacement therapy, substrate reduction therapy, gene therapy, and emerging modalities. Emphasis will be placed on early diagnosis, multidisciplinary care, patient advocacy, and ethical considerations such as equitable treatment access and personalized medicine. Finally, the review will highlight the need for continued research and collaboration to advance understanding and improve therapeutic interventions for individuals with LSDs.

### Significance and novelty of the review

Lysosomal storage diseases (LSDs) represent a complex group of inherited metabolic disorders characterized by the accumulation of undigested macromolecules in the lysosomes due to enzyme deficiencies. These diseases often present with various clinical manifestations, particularly affecting the neurological system^19,20^. The novelty of this review lies in its comprehensive and integrative approach to understanding the neurological manifestations of LSDs, which sets it apart from previous literature in several significant ways^21^. Firstly, this review compiles and analyzes the latest research findings on the neurological manifestations of LSDs^22^. Previous reviews have often focused on individual diseases or a limited subset of LSDs.

In contrast, this review encompasses a broad spectrum of LSDs, providing a more holistic view of their neurological impacts. This inclusive approach not only helps in understanding the commonalities and differences among various LSDs but also aids in identifying potential shared pathophysiological mechanisms^23^.

Moreover, this review emphasizes the emerging diagnostic techniques and therapeutic strategies developed in recent years^23^. Traditional reviews have typically concentrated on established diagnostic criteria and treatment modalities^24^. However, this review highlights novel diagnostic biomarkers and advanced imaging techniques currently under investigation. For instance, recent studies have identified specific biomarkers in cerebrospinal fluid that can aid in the early diagnosis of neurological involvement in LSDs^25^. Additionally, advancements in magnetic resonance imaging (MRI) and other neuroimaging modalities are discussed, which offer better sensitivity and specificity in detecting neurological abnormalities associated with these diseases.

Another unique aspect of this review is its focus on the genetic and molecular underpinnings of the neurological manifestations of LSDs^26^. While earlier literature has acknowledged the genetic basis of LSDs, this review delves deeper into the genetic mutations and molecular pathways that contribute to the neurological symptoms^10^. By integrating findings from genomics and proteomics, the review provides a detailed account of how specific genetic alterations can lead to lysosomal dysfunction and subsequent neurological damage. This molecular perspective enhances our understanding of disease mechanisms and opens new avenues for targeted therapies^11^.

In addition, the review addresses the therapeutic potentials and limitations of current treatment options for neurological manifestations of LSDs. While enzyme replacement therapy (ERT) and hematopoietic stem cell transplantation (HSCT) are well-documented in existing literature, this review also discusses newer approaches such as gene therapy, substrate reduction therapy, and pharmacological chaperones^27^. These emerging treatments hold promise for more effective management of neurological symptoms but also come with challenges. By critically evaluating these therapies, the review provides valuable insights into their feasibility and potential impact on patient outcomes. The review also emphasizes the importance of early diagnosis and intervention in improving neurological outcomes in patients with LSDs. Many of the neurological manifestations of LSDs are progressive and irreversible if not treated promptly^28^. This review synthesizes evidence from recent studies that underscore the benefits of early therapeutic intervention in halting or slowing disease progression. It also highlights the role of newborn screening programs and genetic counseling in facilitating early detection and management of LSDs^12^.

Furthermore, the review provides a comprehensive overview of the clinical presentation and progression of neurological symptoms in LSDs. While previous literature has often described these symptoms fragmentedly, this review systematically categorizes them based on their clinical features and progression^13^. This organized approach helps in identifying patterns and correlations between different types of LSDs and their neurological manifestations^29^. For example, the review discusses how specific LSDs like Gaucher disease and Niemann–Pick disease present distinct neurological phenotypes, thereby aiding in differential diagnosis. Another novel contribution of this review is its emphasis on patient-centered care and quality of life. The impact of neurological symptoms on the daily lives of patients with LSDs is profound, affecting their physical, cognitive, and emotional well-being^14^. This review incorporates patient perspectives and highlights the importance of a multidisciplinary approach in managing these complex conditions. It discusses the role of neurologists, geneticists, physical therapists, and other healthcare professionals in providing comprehensive care to patients with LSDs^15^. In terms of methodology, this review adopts a rigorous and systematic approach to literature selection and analysis. It employs advanced search strategies and inclusion criteria to ensure that the most relevant and high-quality studies are considered^11^. The review also utilizes established tools for assessing the quality and risk of bias in the included studies, thereby enhancing the reliability of its findings. This methodological rigor distinguishes it from previous reviews that may have relied on less systematic approaches^27^. Lastly, the review identifies gaps in current research and suggests directions for future studies. While substantial progress has been made in understanding and managing the neurological manifestations of LSDs, many questions remain unanswered^28^. This review outlines key areas where further research is needed, such as the long-term efficacy of new therapies, developing more sensitive diagnostic tools, and exploring novel molecular targets. By highlighting these research priorities, the review aims to stimulate further investigation and innovation in the field^12^.

## Materials and methods

### Literature review

A structured literature review was conducted to gather information on the neurological manifestations of lysosomal storage diseases (LSDs). The search included multiple databases with tailored keywords for each:

1. *PubMed*: Keywords: “Lysosomal Storage Diseases,” “Neurological Manifestations,” “Cognitive Impairment,” “Motor Dysfunction,” “Seizures,” “Sensory Deficits,” “Psychiatric Symptoms,” “Diagnosis,” “Enzyme Replacement Therapy,” “Gene Therapy,” “Substrate Reduction Therapy,” “Emerging Therapies.”
2. *Embase*: Keywords similar to PubMed, supplemented with Embase-specific indexing terms (Emtree).
3. *Cochrane Library*: Focused on reviews and clinical trials with keywords such as “Lysosomal Storage Diseases” AND “Neurological Symptoms” and terms related to therapeutic interventions.
4. *Scopus*: Combined broader keywords including “Lysosomal Storage Disorders” AND “Neurology.”
5. *Web of Science*: Used similar keywords, focusing on high-impact journal articles.

Boolean operators (AND, OR) were used to refine the searches, ensuring a comprehensive collection of relevant studies. The search was restricted to peer-reviewed articles published in English from January 2000 to June 2024.

#### Inclusion and exclusion criteria

The review included studies and reviews published in peer-reviewed journals, clinical guidelines, and authoritative textbooks. Articles written in English and relevant to the scope of the manuscript were considered. Studies involving animal models or *in vitro* experiments were included if they provided insights into the pathophysiology or treatment of LSDs with neurological involvement.

#### Data extraction

Data were extracted from selected articles, including study design, patient characteristics, neurological manifestations observed, diagnostic approaches, treatment modalities, and outcomes. Key findings and relevant references were compiled for further analysis and discussion.

#### Synthesis of information

The extracted data were synthesized to provide a comprehensive overview of the neurological manifestations of LSDs and their management. Common themes and patterns observed across different LSD subtypes were identified, and gaps in the existing literature were noted.

#### Quality assurance

The quality and validity of the included studies were assessed using the Joanna Briggs Institute (JBI) critical appraisal tools, which are suitable for various study designs^13^.

1. Risk of Bias Assessment Tool:

- The JBI Critical Appraisal Checklists for Systematic Reviews and Research Syntheses, Randomized Controlled Trials, and Cohort Studies were employed. These tools assess criteria such as study design, data collection, and analysis rigor^13^.
- Two reviewers independently evaluated each study, and disagreements were resolved through discussion or consultation with a third reviewer^13^.

## Definition and epidemiology

Lysosomal storage diseases (LSDs) are a group of rare inherited metabolic disorders, collectively affecting an estimated 1 in 5000 to 1 in 10 000 live births worldwide^1^. While individual LSDs are individually rare, collectively, they represent a significant burden on affected individuals, families, and healthcare systems. The prevalence of specific LSDs varies widely, with some disorders being more common in certain populations or ethnic groups. For example, Gaucher disease, the most prevalent LSD, has an estimated incidence of 1 in 40 000–60 000 live births in the general population but may be more common in individuals of Ashkenazi Jewish descent, with an incidence as high as 1 in 850 live births^2^. Similarly, Niemann–Pick disease types A and B have higher incidences in certain populations, such as individuals of Northern European descent, where the combined incidence may be as high as 1 in 50 000 live births^3^.

Conversely, other LSDs, such as Tay–Sachs disease, are more prevalent in specific ethnic groups, such as individuals of Ashkenazi Jewish, French-Canadian, or Cajun descent, with an estimated carrier frequency of 1 in 27 to 1 in 30 individuals in these populations^4^. The incidence and prevalence of LSDs may also be influenced by factors such as consanguinity, genetic heterogeneity, and advances in diagnostic techniques. Improved awareness and access to genetic testing have led to increased detection rates and more accurate estimates of disease prevalence in recent years^30^. Despite their rarity, LSDs pose significant challenges for affected individuals and their families due to the chronic and progressive nature of these disorders, as well as the associated physical, cognitive, and psychosocial disabilities. Early diagnosis and intervention are critical for optimizing outcomes and improving the quality of life for individuals with LSDs and their families^5^. Further research is needed to understand better the epidemiology of LSDs, including the factors contributing to disease prevalence, distribution, and variability across different populations. Enhanced surveillance and screening programs, coupled with advances in genetic and molecular diagnostics, will be essential for improving early detection and intervention for individuals affected by LSDs^6^.

## Etiology and pathophysiology

Lysosomal storage diseases (LSDs) encompass a diverse group of inherited metabolic disorders characterized by the accumulation of undegraded substrates within lysosomes, leading to cellular dysfunction and tissue damage^31^. The etiology of LSDs varies depending on the specific enzyme or protein deficiency involved and the type of substrate that accumulates. However, a common underlying mechanism in LSDs is the impairment of lysosomal function, which disrupts the normal degradation and recycling of cellular macromolecules^7^. Lysosomes are membrane-bound organelles containing a variety of hydrolytic enzymes responsible for breaking down proteins, lipids, carbohydrates, and nucleic acids into their constituent molecules^8^. These enzymes are synthesized in the endoplasmic reticulum and then transported to the Golgi apparatus, where they are modified and packaged into lysosomes for intracellular digestion^9^. Lysosomal enzymes require an acidic environment to function optimally, which is maintained by the activity of proton pumps in the lysosomal membrane^10^. In LSDs, mutations in genes encoding lysosomal enzymes or transport proteins disrupt the normal function of lysosomes, leading to the accumulation of substrates that cannot be adequately degraded^11^. The specific substrate that accumulates varies depending on the enzyme deficiency, but common examples include glycosaminoglycans, sphingolipids, glycoproteins, and glycogen^27^. The accumulation of substrates within lysosomes results in lysosomal enlargement and dysfunction, impairing the normal process of autophagy, which is the cellular mechanism for degrading and recycling damaged or obsolete organelles and macromolecules^28^. The accumulation of undegraded material disrupts cellular homeostasis, leading to cellular stress, inflammation, and ultimately, cell dysfunction and death^12^. The pathophysiology of LSDs is highly variable and can affect multiple organ systems, including the central nervous system (CNS), skeletal system, cardiovascular system, and hematopoietic system^13^. Neurological involvement is a prominent feature of many LSDs and can manifest in various ways, including cognitive impairment, motor dysfunction, seizures, sensory deficits, and psychiatric symptoms^29^. The extent and severity of neurological manifestations in LSDs depend on various factors, including the specific enzyme deficiency, the type of substrate that accumulates, the age of onset, and the rate of disease progression^29^. In some LSDs, such as Tay–Sachs disease and Niemann–Pick disease type C, the accumulation of lipids within neurons leads to progressive neurodegeneration and a wide range of neurological symptoms^15^. In other LSDs, such as Gaucher disease and mucopolysaccharidoses, the neurological manifestations may be secondary to systemic disease processes, such as inflammation, hypoxia, or metabolic disturbances^16^. The blood-brain barrier (BBB) presents a significant challenge for the treatment of neurological manifestations in LSDs, as it restricts the entry of therapeutic agents into the CNS^17^. While some enzyme replacement therapies (ERTs) have been developed to treat LSDs, their efficacy in addressing CNS involvement is limited by the inability of the therapeutic enzyme to cross the BBB^18^. Substrate reduction therapies (SRTs) and gene therapy approaches hold promise for addressing CNS manifestations in LSDs by targeting the underlying molecular defects within affected cells^19^. In addition to the primary enzyme deficiency and substrate accumulation, secondary mechanisms may contribute to the pathophysiology of neurological manifestations in LSDs. These mechanisms include oxidative stress, neuroinflammation, mitochondrial dysfunction, and impaired neurotrophic support^29^. The progressive nature of LSDs further exacerbates these secondary processes, leading to cumulative damage and worsening neurological symptoms over time^14^.

## Neurological manifestations of LSDs

Neurological manifestations are prominent features of lysosomal storage diseases (LSDs), contributing significantly to the morbidity and mortality associated with these disorders^20^. The spectrum of neurological symptoms observed in LSDs is diverse and can affect various aspects of cognitive, motor, sensory, and psychiatric functioning^21^. Understanding the nature and impact of these neurological manifestations is essential for accurate diagnosis, prognosis, and management of individuals with LSDs. Cognitive impairment and developmental delay are common neurological manifestations observed in many LSDs, particularly those affecting the central nervous system (CNS)^18^. These deficits often manifest early in life and can profoundly affect intellectual functioning, learning, and adaptive skills. For example, individuals with Tay–Sachs disease, a severe form of LSD characterized by the accumulation of gangliosides in neurons, typically experience progressive neurodegeneration and cognitive decline, leading to severe intellectual disability and developmental regression^19^.

Similarly, individuals with Niemann–Pick disease type C may exhibit cognitive impairment and developmental delay due to the accumulation of cholesterol and sphingolipids within neurons, affecting synaptic function and neurotransmitter signaling^20^. Motor dysfunction and movement disorders are also prevalent neurological manifestations in LSDs, affecting both voluntary and involuntary movements. Muscle weakness, hypotonia, and spasticity are common features observed in many LSDs, resulting from the progressive degeneration of motor neurons and muscle fibers^21^. Movement disorders such as dystonia, tremors, and ataxia may also occur, reflecting disturbances in basal ganglia and cerebellar function. For example, individuals with Gaucher disease, a lysosomal storage disorder caused by a deficiency of the enzyme glucocerebrosidase, may develop Parkinsonism-like symptoms, including bradykinesia, rigidity, and postural instability, due to the accumulation of glucosylceramide in the basal ganglia^22^. Seizures and epilepsy are significant neurological manifestations observed in several LSDs, affecting individuals of all ages. The underlying mechanisms of epilepsy in LSDs are complex and may involve neuronal hyperexcitability, neurotransmitter imbalances, and structural brain abnormalities^23^. For example, individuals with mucopolysaccharidosis (MPS), a group of LSDs characterized by the accumulation of glycosaminoglycans, may experience seizures due to cortical and subcortical neuronal dysfunction caused by the abnormal storage of substrates within neurons and glial cells^24^.

Similarly, individuals with neuronal ceroid lipofuscinoses (NCLs), a group of LSDs characterized by the accumulation of lipofuscin within neurons, may develop epilepsy as a result of progressive neuronal loss and gliosis in the cerebral cortex and subcortical structures^8^. Sensory deficits and neuropathic pain are common neurological manifestations in LSDs, affecting sensory perception and processing. Peripheral neuropathy, characterized by numbness, tingling, and loss of sensation, may occur in individuals with LSDs due to the progressive degeneration of peripheral nerves and sensory neurons^9^. Neuropathic pain, described as burning, shooting, or stabbing sensations, may also accompany sensory deficits and contribute to the overall burden of disease. For example, individuals with Fabry disease, a lysosomal storage disorder caused by a deficiency of the enzyme alpha-galactosidase A, often experience neuropathic pain due to the accumulation of globotriaosylceramide in sensory neurons and small nerve fibers^10^. Psychiatric symptoms and behavioral abnormalities are frequently observed in individuals with LSDs, affecting mood, cognition, and social functioning. Depression, anxiety, and psychosis may occur as a result of CNS involvement, neurotransmitter imbalances, and psychosocial stressors associated with living with a chronic and progressive disease^11^. Behavioral disturbances such as aggression, impulsivity, and disinhibition may also manifest, reflecting disturbances in frontal lobe function and executive control. For example, individuals with Sanfilippo syndrome, a severe form of MPS, may exhibit hyperactivity, aggression, and self-injurious behavior due to progressive neurodegeneration and loss of inhibitory control^27^. Table 1 illustrates the various neurological symptoms, diagnostic biomarkers, imaging findings, genetic mutations, available therapies, therapeutic limitations, and research gaps associated with different lysosomal storage diseases.

**Table 1:** Summary of neurological manifestations in lysosomal storage diseases.

Disease	Neurological symptoms	Diagnostic biomarkers	Imaging findings	Genetic mutations	Available therapies	Therapeutic limitations	Research gaps
Gaucher disease	Parkinsonism, cognitive impairment, myoclonus	Glucocerebrosidase enzyme levels	Brain MRI showing white matter changes	GBA1	Enzyme replacement therapy (ERT), substrate reduction therapy (SRT)	Limited efficacy in CNS symptoms	Long-term effects of new therapies
Fabry disease	Stroke, peripheral neuropathy, hearing loss	Alpha-galactosidase A enzyme levels	MRI showing stroke-like lesions, angiokeratomas	GLA	ERT, SRT, gene therapy	Variability in patient response	Impact of early intervention
Niemann–Pick disease	Ataxia, vertical supranuclear gaze palsy, dystonia	Acid sphingomyelinase activity, cholesterol levels	MRI showing cerebellar atrophy	SMPD1 (type A/B), NPC1, NPC2 (type C)	ERT, miglustat (SRT)	Limited penetration to CNS	Efficacy of combination therapies
Tay–Sachs disease	Developmental delay, seizures, vision loss	Hexosaminidase A enzyme levels	MRI showing white matter changes	HEXA	Supportive care, investigational therapies	Lack of curative treatments	Development of gene therapies
Krabbe disease	Irritability, spasticity, developmental delay	Galactocerebrosidase activity	MRI showing demyelination	GALC	HSCT, investigational gene therapy	High mortality despite treatment	Long-term outcomes post-HSCT
Metachromatic leukodystrophy	Progressive motor and cognitive decline	Arylsulfatase A activity	MRI showing white matter changes	ARSA	HSCT, investigational enzyme therapy	Limited options for symptomatic patients	Role of early HSCT
Mucopolysaccharidosis	Cognitive decline, hydrocephalus, spinal cord compression	Glycosaminoglycans levels	MRI showing dysostosis multiplex	Multiple genes (e.g. IDUA, IDS)	ERT, HSCT, gene therapy	Limited efficacy for CNS symptoms	Improved CNS-targeted therapies
Pompe disease	Muscle weakness, respiratory distress, feeding difficulties	Acid alpha-glucosidase levels	MRI showing muscle atrophy	GAA	ERT, gene therapy	Limited long-term efficacy	New therapeutic strategies

Table 1 covers various lysosomal storage diseases (LSDs), highlighting their neurological symptoms, diagnostic biomarkers, imaging findings, genetic mutations, available therapies, therapeutic limitations, and research gaps. Lysosomal storage diseases manifest a wide range of neurological symptoms, such as cognitive decline, motor dysfunction, seizures, and sensory deficits. Diagnostic biomarkers often include specific enzyme deficiencies detectable through biochemical assays or abnormal metabolite levels in blood and urine. Imaging findings frequently reveal characteristic changes, such as brain atrophy, white matter abnormalities, or specific patterns of substrate accumulation visible on MRI or other imaging modalities. Genetic mutations responsible for these diseases are diverse, affecting genes encoding lysosomal enzymes or transport proteins. For instance, Tay–Sachs disease is caused by mutations in the HEXA gene, while Gaucher disease results from mutations in the GBA gene. Available therapies vary by disease but commonly include enzyme replacement therapy (ERT) and substrate reduction therapy (SRT). For example, ERT is a standard treatment for Gaucher and Pompe diseases. However, these therapies often face limitations, such as poor penetration across the blood-brain barrier, limiting their effectiveness in treating neurological symptoms. Therapeutic limitations also arise from the variable response to treatment, the risk of immune reactions, and the high cost of therapies. Despite advances, there are significant research gaps, including the need for therapies that effectively target central nervous system involvement and a better understanding of the natural history and progression of neurological symptoms in these disorders. Emerging treatments, such as gene therapy and pharmacological chaperones, are under investigation but require further research to establish their efficacy and safety. Addressing these gaps is crucial for improving outcomes and quality of life for patients with LSDs.

## CNS involvement in specific lysosomal storage diseases

*Tay–Sachs disease (TSD)* is a rare, inherited lysosomal storage disorder characterized by progressive neurodegeneration and severe neurological impairment, primarily affecting infants and young children. It is caused by mutations in the HEXA gene, resulting in deficient activity of the hexosaminidase A (HexA) enzyme. Without functional HexA, gangliosides, particularly GM2 ganglioside, accumulate within the lysosomes of neurons, leading to cellular dysfunction, neuroinflammation, and, ultimately, neuronal death^1^. CNS involvement in TSD is profound and pervasive, with neurological manifestations typically becoming apparent in early infancy. Infants with TSD typically present with developmental regression, loss of motor skills, and hypotonia within the first few months of life. As the disease progresses, affected individuals may develop spasticity, seizures, and feeding difficulties, eventually leading to profound cognitive impairment and paralysis^2^. Neuroimaging studies in individuals with TSD reveal characteristic abnormalities, including cortical atrophy, enlargement of the lateral ventricles, and hypodensities in the basal ganglia and thalamus. These structural changes reflect the widespread neurodegeneration and loss of gray matter observed in TSD^3^. Magnetic resonance spectroscopy studies have also demonstrated elevated neuronal metabolites such as *N*-acetyl aspartate (NAA) and decreased creatine levels, reflecting neuronal loss and dysfunction in affected individuals^4^. Histopathological examination of the brain in TSD reveals widespread neuronal loss, gliosis, and the presence of storage material, particularly in neurons of the cerebral cortex, brainstem, and spinal cord. In addition to neurons, glial cells, including astrocytes and oligodendrocytes, may also contain storage material, contributing to neuroinflammation and progressive neurodegeneration^30^. The clinical course of TSD is relentless, with affected individuals typically succumbing to the disease by early childhood. However, variants of TSD, including late-onset and adult-onset forms, have been reported with milder phenotypes and slower disease progression. In these cases, CNS involvement may manifest later in life, with symptoms such as progressive cognitive decline, motor dysfunction, and psychiatric symptoms^5^. Management of CNS involvement in TSD primarily focuses on supportive care and symptomatic management, as there is currently no cure for the disease. Early intervention with supportive therapies such as physical, occupational, and speech therapy can help optimize the quality of life and functional outcomes for affected individuals. Additionally, seizure management with antiepileptic medications and nutritional support may be necessary to address specific symptoms and complications associated with CNS involvement^6^. Emerging therapeutic approaches for TSD aim to address the underlying pathophysiology of the disease, including enzyme replacement therapy (ERT) and gene therapy. ERT involves administering recombinant HexA enzyme to replace the deficient enzyme activity in affected individuals. While ERT has shown promise in preclinical studies, challenges such as blood-brain barrier penetration and immune responses to the exogenous enzyme remain significant hurdles to overcome^31^. Gene therapy approaches for TSD involve the delivery of functional copies of the HEXA gene to affected cells, either through viral vectors or genome editing techniques such as CRISPR-Cas9. Preclinical studies in animal models have demonstrated the potential efficacy of gene therapy in correcting the underlying genetic defect and preventing neurodegeneration in TSD^7^.

*Gaucher disease (GD)* is an autosomal recessive lysosomal storage disorder caused by deficient activity of the enzyme glucocerebrosidase (GCase), leading to the accumulation of glucosylceramide primarily within lysosomes of macrophages, known as Gaucher cells^1^. While Gaucher’s disease primarily affects the reticuloendothelial system, CNS involvement is a significant aspect of the disease, contributing to a wide range of neurological manifestations and complications^2^. CNS involvement in Gaucher disease can manifest in various forms, including cognitive impairment, movement disorders, seizures, and peripheral neuropathy^3^. The specific neurological manifestations observed in Gaucher disease vary depending on the subtype of the disease, the age of onset, and the severity of enzyme deficiency^4^. In general, CNS involvement is more common and severe in individuals with neuronopathic forms of Gaucher disease, such as type 2 and type 3 GD, compared to non-neuronopathic forms, such as type 1 GD^30^. One of the hallmark neurological manifestations of Gaucher disease is cognitive impairment, which can range from mild deficits in executive function and attention to severe intellectual disability and dementia^5^. Cognitive impairment in Gaucher disease is believed to result from the accumulation of glucosylceramide within neurons and glial cells, leading to neuroinflammation, synaptic dysfunction, and progressive neurodegeneration^6^. Neuroimaging studies in individuals with Gaucher disease reveal structural abnormalities, including cortical atrophy, white matter changes, and alterations in brain volume, particularly in regions involved in memory, executive function, and motor control^31^. Movement disorders are also common neurological manifestations of Gaucher disease, affecting voluntary and involuntary movements. Parkinsonism-like symptoms, including bradykinesia, rigidity, and resting tremor, may occur in individuals with Gaucher disease, particularly in the context of significant CNS involvement^7^. The pathophysiology of movement disorders in Gaucher disease is complex and multifactorial, involving dysfunction of the basal ganglia, disruption of dopamine signaling, and neuroinflammation^8^. Additionally, dystonia, chorea, and ataxia may occur in individuals with more severe forms of Gaucher disease, reflecting disturbances in cerebellar and extrapyramidal pathways^9^. Seizures are another neurological complication observed in Gaucher disease, particularly in individuals with neuronopathic forms of the disease. The mechanisms underlying seizure development in Gaucher disease are not fully understood but may involve alterations in neuronal excitability, neurotransmitter imbalances, and structural brain abnormalities^10^. Seizures in Gaucher disease can be challenging to manage and may require treatment with antiepileptic medications, seizure precautions, and close monitoring for complications such as status epilepticus and cognitive decline^11^. Peripheral neuropathy is a common neurological manifestation observed in Gaucher disease, affecting sensory, motor, and autonomic nerve fibers. Peripheral neuropathy in Gaucher disease is believed to result from the accumulation of glucosylceramide within Schwann cells and peripheral nerves, leading to demyelination, axonal degeneration, and impaired nerve conduction^27^. Clinical features of peripheral neuropathy in Gaucher disease may include numbness, tingling, weakness, and sensory loss, particularly in the distal extremities^28^. Management of peripheral neuropathy in Gaucher disease typically involves symptomatic treatment with analgesic medications, physical therapy, and supportive care to optimize functional outcomes and quality of life^12^. The pathophysiology of CNS involvement in Gaucher disease is complex and multifactorial, involving disruptions in lysosomal function, accumulation of glucosylceramide within neurons and glial cells, neuroinflammation, and progressive neurodegeneration^13^. Animal models of Gaucher disease have provided valuable insights into the underlying mechanisms of CNS involvement and have been instrumental in developing therapeutic strategies to mitigate neurological manifestations^29^. Management of CNS involvement in Gaucher disease primarily focuses on supportive care and symptomatic management, as there is currently no cure for the disease. Enzyme replacement therapy (ERT) and substrate reduction therapy (SRT) are the mainstay of treatment for Gaucher disease, aimed at reducing the burden of glucosylceramide accumulation and mitigating disease progression^14^. ERT involves administering recombinant GCase enzyme to replace the deficient enzyme activity in affected individuals, while SRT involves using small-molecule inhibitors to reduce the production of glucosylceramide^15^.

*Niemann–Pick disease (NPD)* encompasses a group of rare, inherited lysosomal storage disorders caused by mutations in genes encoding proteins involved in lipid metabolism, resulting in the accumulation of sphingomyelin and cholesterol within lysosomes. This accumulation leads to cellular dysfunction, inflammation, and progressive neurodegeneration, contributing to the diverse clinical manifestations observed in affected individuals^1^. Central nervous system (CNS) involvement is a prominent feature of Niemann–Pick disease, with neurological manifestations typically becoming apparent in early infancy or childhood. The severity and spectrum of CNS involvement vary depending on the subtype of Niemann–Pick disease, the age of onset, and the specific genetic mutation involved^2^. Several subtypes of Niemann–Pick disease, including types A, B, and C, have distinct clinical presentations and neurological features. Niemann–Pick disease type A (NPA) is the most severe form of the disease, characterized by deficient activity of the enzyme acid sphingomyelinase (ASM), leading to the accumulation of sphingomyelin primarily within lysosomes of macrophages. CNS involvement in NPA is profound and progressive, with affected individuals typically presenting with developmental delay, hypotonia, hepatosplenomegaly, and progressive neurodegeneration^3^. Neurological manifestations of NPA include loss of motor skills, seizures, hypertonia, and impaired feeding, reflecting the widespread neuroinflammation and neuronal dysfunction observed in affected individuals^4^. Niemann–Pick disease type B (NPB) is a milder form of the disease, characterized by deficient activity of ASM and the accumulation of sphingomyelin primarily within the lysosomes of macrophages. While NPB primarily affects the reticuloendothelial system, CNS involvement can occur, particularly in individuals with a severe enzyme deficiency or certain genetic mutations^30^. Neurological manifestations of NPB may include cognitive impairment, developmental delay, and peripheral neuropathy, although these symptoms are typically less severe than those observed in NPA^5^. Additionally, individuals with NPB may present with hepatosplenomegaly, pulmonary involvement, and bone abnormalities, reflecting the systemic nature of the disease^6^. Niemann–Pick disease type C (NPC) is a distinct subtype of the disease caused by mutations in the NPC1 or NPC2 gene. This leads to impaired intracellular trafficking and the accumulation of cholesterol and glycosphingolipids within lysosomes. NPC is characterized by a wide range of neurological manifestations, including cognitive impairment, movement disorders, seizures, and progressive neurodegeneration^31^. The age of onset and severity of symptoms in NPC vary widely, with some individuals presenting in early infancy with severe neurological impairment. In contrast, others may present in adulthood with milder cognitive and motor dysfunction^7^. CNS involvement in NPC is complex and multifactorial, involving lipid metabolism disruptions, neuronal signaling alterations, and neuroinflammation. The accumulation of cholesterol and glycosphingolipids within neurons and glial cells leads to cellular dysfunction and progressive neurodegeneration, particularly in regions of the brain involved in memory, cognition, and motor control^8^. Neuroimaging studies in individuals with NPC reveal characteristic abnormalities, including white matter changes, cortical atrophy, and alterations in brain volume, reflecting the widespread neurodegeneration observed in affected individuals^9^. In addition to the hallmark neurological manifestations, individuals with Niemann–Pick disease may also present with ophthalmological abnormalities, including cherry-red spots on the macula, vertical gaze palsy, and optic atrophy. These ocular findings indicate neuronal storage and neurodegeneration within the retina and optic nerve and may aid in diagnosing and monitoring disease progression^10^. Management of CNS involvement in Niemann–Pick disease primarily focuses on supportive care and symptomatic management, as there is currently no cure for the disease. Early intervention with supportive therapies such as physical, occupational, and speech therapy can help optimize the quality of life and functional outcomes for affected individuals. Additionally, seizure management with antiepileptic medications and nutritional support may be necessary to address specific symptoms and complications associated with CNS involvement^11^. Emerging therapeutic approaches for Niemann–Pick disease aim to address the underlying pathophysiology of the disease, including substrate reduction therapy (SRT), gene therapy, and pharmacological chaperone therapy. SRT involves using small-molecule inhibitors to reduce the production of sphingomyelin or cholesterol, thereby reducing the burden of substrate accumulation and mitigating disease progression^27^. Gene therapy approaches for Niemann–Pick disease involve the delivery of functional copies of the affected gene to affected cells, either through viral vectors or genome editing techniques such as CRISPR-Cas9. Preclinical studies in animal models have shown promising results, highlighting the potential efficacy of gene therapy in correcting the underlying genetic defect and preventing neurodegeneration in Niemann–Pick disease^28^.

*Pompe disease, or glycogen storage disease type II*, is a rare autosomal recessive lysosomal storage disorder caused by mutations in the gene encoding acid alpha-glucosidase (GAA), an enzyme responsible for breaking down glycogen into glucose within lysosomes. Deficiency or complete absence of GAA activity results in glycogen accumulation, primarily within lysosomes of skeletal and cardiac muscle cells, leading to cellular dysfunction, inflammation, and progressive organ damage^1^. While Pompe disease primarily affects skeletal and cardiac muscle, CNS involvement can occur, particularly in the infantile-onset form of the disease. The severity and spectrum of CNS involvement in Pompe disease vary depending on the age of onset, the extent of enzyme deficiency, and the specific genetic mutation involved^2^. Infantile-onset Pompe disease is the most severe form of the disease, typically presenting within the first few months of life with hypotonia, muscle weakness, cardiomegaly, and respiratory distress. CNS involvement in infantile-onset Pompe disease is profound and progressive, with affected individuals demonstrating developmental delay, cognitive impairment, and neurologic regression^3^. The pathophysiology of CNS involvement in Pompe disease is complex and multifactorial, involving disruptions in glycogen metabolism, alterations in neuronal signaling, and neuroinflammation. The accumulation of glycogen within neurons and glial cells leads to cellular dysfunction and progressive neurodegeneration, particularly in brain regions involved in motor control, cognition, and autonomic function^4^. Neuroimaging studies in individuals with Pompe disease reveal characteristic abnormalities, including white matter changes, cortical atrophy, and alterations in brain volume, reflecting the widespread neurodegeneration observed in affected individuals. Additionally, magnetic resonance spectroscopy studies have demonstrated altered levels of neuronal metabolites such as *N*-acetyl aspartate (NAA) and creatine, reflecting neuronal loss and dysfunction in affected individuals^30^. In addition to the hallmark neurological manifestations, individuals with Pompe disease may also present with ophthalmological abnormalities, including ptosis, ophthalmoplegia, and retinal degeneration. These ocular findings indicate neuronal storage and neurodegeneration within the extraocular muscles and may aid in diagnosing and monitoring disease progression^5^. Management of CNS involvement in Pompe disease primarily focuses on supportive care and symptomatic management, as there is currently no cure for the disease. Early intervention with supportive therapies such as physical, occupational, and speech therapy can help optimize the quality of life and functional outcomes for affected individuals. Additionally, seizure management with antiepileptic medications and nutritional support may be necessary to address specific symptoms and complications associated with CNS involvement^6^. Emerging therapeutic approaches for Pompe disease aim to address the underlying pathophysiology of the disease, including enzyme replacement therapy (ERT), gene therapy, and pharmacological chaperone therapy. ERT involves administering recombinant GAA enzyme to replace the deficient enzyme activity in affected individuals, thereby reducing the burden of glycogen accumulation and mitigating disease progression^31^. Gene therapy approaches for Pompe disease involve the delivery of functional copies of the GAA gene to affected cells, either through viral vectors or genome editing techniques such as CRISPR-Cas9. Preclinical studies in animal models have shown promising results, highlighting the potential efficacy of gene therapy in correcting the underlying genetic defect and preventing neurodegeneration in Pompe disease^7^.

*Mucopolysaccharidoses (MPS)* are a group of inherited lysosomal storage disorders characterized by the deficiency of enzymes responsible for the degradation of glycosaminoglycans (GAGs), leading to their accumulation within lysosomes and extracellular matrix. The progressive accumulation of GAGs within cells and tissues results in cellular dysfunction, inflammation, and progressive organ damage, contributing to the diverse clinical manifestations observed in affected individuals^1^. Central nervous system (CNS) involvement is a prominent feature of many MPS disorders, contributing to the neurological manifestations and cognitive impairment observed in affected individuals. The severity and spectrum of CNS involvement vary depending on the subtype of MPS, the age of onset, and the specific genetic mutation involved^2^. Individuals with MPS may present with developmental delay, cognitive impairment, and behavioral abnormalities, reflecting the widespread neuroinflammation and progressive neurodegeneration observed in affected individuals. The accumulation of GAGs within neurons and glial cells leads to cellular dysfunction, disruption of neuronal signaling, and progressive neurodegeneration, particularly in regions of the brain involved in memory, cognition, and motor control^3^. Neuroimaging studies in individuals with MPS reveal characteristic abnormalities, including white matter changes, cortical atrophy, and alterations in brain volume, reflecting the widespread neurodegeneration observed in affected individuals. Additionally, magnetic resonance spectroscopy studies have demonstrated altered levels of neuronal metabolites such as *N*-acetyl aspartate (NAA) and creatine, reflecting neuronal loss and dysfunction in affected individuals^4^. In addition to the hallmark neurological manifestations, individuals with MPS may also present with ophthalmological abnormalities, including corneal clouding, retinal degeneration, and optic nerve atrophy. These ocular findings indicate neuronal storage and neurodegeneration within the retina and optic nerve and may aid in diagnosing and monitoring disease progression^30^. Management of CNS involvement in MPS primarily focuses on supportive care and symptomatic management, as there is currently no cure for the disease. Early intervention with supportive therapies such as physical, occupational, and speech therapy can help optimize the quality of life and functional outcomes for affected individuals. Additionally, seizure management with antiepileptic medications and nutritional support may be necessary to address specific symptoms and complications associated with CNS involvement^5^. Emerging therapeutic approaches for MPS aim to address the underlying pathophysiology of the disease, including enzyme replacement therapy (ERT), gene therapy, and substrate reduction therapy (SRT). ERT involves administering recombinant enzymes to replace the deficient enzyme activity in affected individuals, thereby reducing the burden of substrate accumulation and mitigating disease progression^6^. Gene therapy approaches for MPS involve the delivery of functional copies of the affected gene to affected cells, either through viral vectors or genome editing techniques such as CRISPR-Cas9. Preclinical studies in animal models have shown promising results, highlighting the potential efficacy of gene therapy in correcting the underlying genetic defect and preventing neurodegeneration in MPS^31^. Substrate reduction therapy (SRT) involves using small-molecule inhibitors to reduce the production of GAGs, thereby reducing the burden of substrate accumulation and mitigating disease progression^7^.

While many LSDs primarily affect peripheral organs and tissues, several disorders are associated with significant central nervous system (CNS) involvement, contributing to a wide range of neurological manifestations and complications^1^. One example of an LSD with significant CNS involvement is Krabbe disease, also known as globoid cell leukodystrophy. Krabbe disease is caused by mutations in the GALC gene, leading to deficient activity of the enzyme galactocerebrosidase (GALC), which is responsible for the degradation of galactolipids in myelin. The accumulation of galactolipids within oligodendrocytes and Schwann cells leads to demyelination and progressive neurodegeneration, particularly affecting the white matter of the brain and spinal cord. Clinical manifestations of Krabbe disease include developmental delay, motor dysfunction, seizures, and optic nerve atrophy, reflecting the widespread CNS involvement observed in affected individuals^2^. Another LSD with significant CNS involvement is metachromatic leukodystrophy (MLD), which is caused by mutations in the ARSA gene, leading to deficient activity of the enzyme arylsulfatase A (ARSA). ARSA is responsible for the degradation of sulfatides in myelin, and its deficiency results in the accumulation of sulfatides within oligodendrocytes and Schwann cells, leading to demyelination and progressive neurodegeneration. Clinical manifestations of MLD include developmental regression, motor dysfunction, seizures, and peripheral neuropathy, reflecting the widespread CNS involvement observed in affected individuals^3^. Niemann–Pick disease type C (NPC) is another LSD associated with significant CNS involvement caused by mutations in the NPC1 or NPC2 gene, leading to impaired intracellular trafficking and the accumulation of cholesterol and glycosphingolipids within lysosomes. The accumulation of lipids within neurons and glial cells leads to cellular dysfunction and progressive neurodegeneration, particularly affecting brain regions involved in memory, cognition, and motor control. Clinical manifestations of NPC include developmental delay, cognitive impairment, movement disorders, seizures, and progressive neurodegeneration, reflecting the widespread CNS involvement observed in affected individuals^4^. Farber disease is an LSD characterized by deficient activity of the enzyme ceramidase, leading to the accumulation of ceramide within lysosomes and extracellular spaces. While Farber disease primarily affects peripheral tissues, CNS involvement can occur, particularly in severe cases. Clinical manifestations of Farber disease may include developmental delay, cognitive impairment, movement disorders, and peripheral neuropathy, reflecting the widespread CNS involvement observed in affected individuals^30^. Sandhoff disease is an LSD caused by mutations in the HEXB gene, resulting in deficient activity of the enzyme beta-hexosaminidase. The accumulation of GM2 ganglioside within lysosomes leads to cellular dysfunction and progressive neurodegeneration, particularly affecting brain regions involved in motor control and coordination. Clinical manifestations of Sandhoff disease include developmental delay, motor dysfunction, seizures, and progressive neurodegeneration, reflecting the widespread CNS involvement observed in affected individuals^5^. Mucolipidosis type IV (MLIV) is an LSD characterized by deficient activity of the enzyme mucolipin-1, leading to impaired lysosomal trafficking and the accumulation of lipids and other macromolecules within lysosomes. While MLIV primarily affects peripheral tissues, CNS involvement can occur, particularly in severe cases. Clinical manifestations of MLIV may include developmental delay, cognitive impairment, movement disorders, and peripheral neuropathy, reflecting the widespread CNS involvement observed in affected individuals^6^.

## Diagnosis

The history and physical examination play a crucial role in evaluating and diagnosing lysosomal storage diseases (LSDs), aiding clinicians in identifying potential symptoms, assessing disease severity, and guiding further diagnostic workup^1^. Taking a detailed medical history and conducting a thorough physical examination can provide valuable insights into the patient’s presenting symptoms, family history, and overall clinical status, helping to guide the differential diagnosis and management plan. The medical history should begin with a comprehensive review of the patient’s presenting symptoms, including any neurological, developmental, systemic, or multisystemic manifestations suggestive of LSDs. Common neurological symptoms observed in LSDs include cognitive impairment, motor dysfunction, seizures, sensory deficits, and psychiatric symptoms^2^. Developmental delay, regression, and failure to thrive may also be prominent features, particularly in early-onset forms of LSDs^3^. A detailed family history is essential, as many LSDs have a genetic basis and may be inherited in an autosomal recessive or X-linked manner^4^. Inquiring about consanguinity, previous affected family members, and known carrier status can provide valuable information regarding the likelihood of an inherited metabolic disorder. Additionally, a history of genetic testing or prenatal screening for LSDs should be explored if available. Social history factors such as parental consanguinity, ethnicity, and geographic location may also be relevant, as certain LSDs have higher incidences or carrier frequencies in specific populations or ethnic groups^30^. Environmental exposures, dietary habits, and prenatal or perinatal events should be considered, as they may contribute to disease presentation or progression. A thorough physical examination should be conducted to assess the patient’s overall clinical status and identify any signs suggestive of LSDs or associated complications. Neurological examination findings such as hypotonia, hyperreflexia, abnormal movements, and sensory deficits should be carefully documented, as they may provide clues to the underlying etiology and severity of neurological involvement^5^. Dysmorphic features, organomegaly, skeletal abnormalities, and skin lesions may be present in some LSDs and should be systematically evaluated during the physical examination^6^. Hepatosplenomegaly, characteristic facial features (e.g. coarse facies, hypertelorism), skeletal deformities (e.g. kyphosis, scoliosis), and skin findings (e.g. angiokeratomas, cafe-au-lait spots) may raise suspicion for specific LSDs such as Gaucher disease, mucopolysaccharidoses, or Fabry disease, respectively. Assessment of growth parameters such as weight, height, and head circumference is essential, particularly in pediatric patients, as growth failure and developmental delay may be early manifestations of LSDs^31^. Monitoring growth velocity over time can help identify progressive deterioration or regression in growth parameters, which may indicate worsening disease severity or complications. Evaluation of systemic symptoms such as respiratory distress, cardiovascular abnormalities, gastrointestinal dysfunction, and hematological abnormalities should also be included in the physical examination, as LSDs can affect multiple organ systems^7^. Cardiopulmonary auscultation, abdominal palpation, and examination of the skin, mucous membranes, and extremities can provide valuable information regarding the patient’s overall clinical status and help guide further diagnostic evaluation. In addition to the history and physical examination, ancillary studies such as laboratory tests, imaging studies, and genetic testing are often necessary to confirm the diagnosis of LSDs and characterize disease severity^8^. Laboratory tests such as complete blood count, serum chemistries, urinalysis, and specific enzyme assays can provide valuable information regarding metabolic abnormalities, organ dysfunction, and biomarkers of disease activity^9^. Imaging studies such as skeletal radiographs, echocardiography, and neuroimaging (e.g. magnetic resonance imaging, computed tomography) may be indicated to assess for skeletal abnormalities, cardiac involvement, and CNS pathology, respectively^10^. Genetic testing, including targeted mutation analysis, chromosomal microarray, or whole exome sequencing, can help identify the underlying genetic mutation responsible for the LSD and confirm the diagnosis^11^. Magnetic resonance imaging (MRI) is the cornerstone of neuroimaging in LSDs, offering high-resolution images of the brain and spinal cord without exposing individuals to ionizing radiation. Conventional MRI sequences such as T1-weighted, T2-weighted, fluid-attenuated inversion recovery (FLAIR), and proton density images provide detailed anatomical information and enable the detection of structural abnormalities such as cortical atrophy, ventricular enlargement, and gray matter lesions. T1-weighted images highlight the contrast between gray and white matter, facilitating the visualization of cortical and subcortical structures. T2-weighted and FLAIR images are sensitive to white matter pathology, allowing for the detection of hyperintense lesions indicative of demyelination, gliosis, and edema. Proton density images provide additional contrast between tissues, aiding in identifying subtle abnormalities not readily visible on other sequences^1^. Advanced MRI techniques offer additional insights into CNS involvement in LSDs, providing quantitative measures of tissue integrity, metabolic activity, and neuronal function. Magnetic resonance spectroscopy (MRS) enables the non-invasive assessment of neurochemical profiles within specific brain regions, allowing for the measurement of metabolites such as *N*-acetyl aspartate (NAA), creatine (Cr), choline (Cho), and myoinositol (mI). Alterations in metabolite concentrations can provide valuable insights into neuronal loss, axonal damage, and neuroinflammation, aiding in disease severity and progression characterization. Diffusion-weighted imaging (DWI) and diffusion tensor imaging (DTI) assess the microstructural integrity of white matter tracts, allowing for the detection of abnormalities such as reduced fractional anisotropy (FA), increased mean diffusivity (MD) and altered diffusivity patterns indicative of axonal injury, demyelination, and gliosis^2^. Functional MRI (fMRI) assesses regional brain activity and connectivity patterns in response to cognitive tasks or sensory stimuli, providing insights into neuronal function and network organization. Task-based fMRI studies involve the presentation of stimuli or tasks designed to elicit specific cognitive processes or sensory responses. In contrast, resting-state fMRI (rs-fMRI) examines spontaneous fluctuations in blood oxygen level-dependent (BOLD) signals during rest. Alterations in fMRI activation patterns and connectivity networks can provide valuable insights into cognitive impairment, sensorimotor deficits, and alterations in brain network organization associated with CNS involvement in LSDs^3^. Positron emission tomography (PET) and single-photon emission computed tomography (SPECT) are nuclear medicine imaging techniques that assess regional cerebral blood flow, metabolism, and neurotransmitter function *in vivo*. PET imaging using radiotracers such as fluorodeoxyglucose (FDG) or amyloid-beta ligands enables the assessment of glucose metabolism, amyloid deposition, and tau pathology in the brain. SPECT imaging using radiotracers such as technetium-99m (Tc-99m) or iodine-123 (I-123) enables the assessment of regional cerebral blood flow, dopamine transporter density, and neurotransmitter receptor binding. PET and SPECT imaging provide complementary information to MRI and MRS, aiding in the characterization of functional deficits, neurotransmitter abnormalities, and neurodegenerative changes associated with CNS involvement in LSDs^4^. Despite the advances in neuroimaging techniques, several challenges remain in the evaluation of CNS involvement in LSDs. Variability in imaging protocols, equipment, and interpretation standards can affect the consistency and reproducibility of imaging findings across different centers. Additionally, the interpretation of imaging findings requires expertise in neuroanatomy, neuropathology, and neuroimaging techniques, highlighting the importance of multidisciplinary collaboration in the diagnosis and management of LSDs. Future research efforts aimed at standardizing imaging protocols, developing quantitative imaging biomarkers, and integrating multimodal imaging techniques hold promise for improving the accuracy and utility of neuroimaging in evaluating CNS involvement in LSDs^30^. Biomarkers are measurable indicators of biological processes or disease states that can be detected in various biological samples, including blood, urine, cerebrospinal fluid (CSF), and tissues. In LSDs, biomarkers serve as surrogate markers of lysosomal dysfunction, substrate accumulation, neuroinflammation, and neuronal damage, reflecting the underlying pathophysiology of the disease. Biomarkers such as lysosomal enzyme activities, substrate levels, cytokine profiles, and neurochemical concentrations provide valuable insights into disease severity, progression, and response to therapy^1^. Enzyme activity assays are widely used biomarkers for diagnosing and monitoring LSDs, providing quantitative measures of lysosomal enzyme function in affected individuals. Enzyme assays are performed on peripheral blood leukocytes, cultured fibroblasts, or dried blood spots using fluorogenic or chromogenic substrates specific to the enzyme of interest. Reduced enzyme activity levels indicate enzyme deficiency and substrate accumulation, confirming the diagnosis of specific LSDs and monitoring treatment response over time. Enzyme activity assays are particularly useful for assessing the efficacy of enzyme replacement therapy (ERT), substrate reduction therapy (SRT), and other targeted therapies aimed at restoring enzyme activity and reducing substrate accumulation^2^. Substrate quantification assays measure the levels of specific substrates or metabolites associated with lysosomal storage in biological samples such as blood, urine, or CSF. Elevated substrate levels reflect lysosomal dysfunction and substrate accumulation, providing valuable insights into disease severity, progression, and response to therapy. Substrate quantification assays are used to monitor disease progression, assess treatment response, and adjust therapeutic interventions based on changes in substrate levels over time. Biomarkers such as urinary glycosaminoglycans, oligosaccharides, and sulfatides are commonly measured to evaluate lysosomal storage and disease burden in LSDs^3^. Cytokine profiling is a valuable biomarker tool for assessing neuroinflammation and immune dysregulation in LSDs, reflecting the underlying inflammatory processes associated with CNS involvement. Cytokines such as interleukins, tumor necrosis factor-alpha (TNF-α), and interferon-gamma (IFN-γ) mediate neuroinflammation, gliosis, and neuronal damage in affected individuals. Dysregulation of cytokine signaling pathways contributes to disease pathogenesis and exacerbates neurodegeneration in LSDs. Cytokine profiling provides insights into disease mechanisms, identifies potential therapeutic targets, and monitors the efficacy of anti-inflammatory interventions in LSDs^4^. Neurochemical analysis using techniques such as mass spectrometry and chromatography enables the quantification of neurotransmitters, metabolites, and neuronal markers in biological samples. Neurochemical alterations reflect neuronal dysfunction, axonal damage, and neurotransmitter imbalances associated with CNS involvement in LSDs. Biomarkers such as *N*-acetyl aspartate (NAA), creatine (Cr), choline (Cho), and myoinositol (mI) provide valuable insights into neuronal integrity, metabolic activity, and glial cell activation in affected individuals. Neurochemical analysis characterizes disease severity, progression, and treatment response, guiding therapeutic interventions to preserve neuronal function and mitigate neurodegeneration^30^. Long-term monitoring and disease progression are essential aspects of managing neurological manifestations in LSDs, as the natural history of these disorders is characterized by progressive neurodegeneration and functional decline over time. Regular clinical assessments, biomarker analyses, neuroimaging studies, and functional evaluations are necessary to track disease progression, evaluate treatment response, and adjust therapeutic interventions accordingly. Longitudinal studies and disease registries play a crucial role in collecting longitudinal data on disease outcomes, natural history, and treatment efficacy, providing valuable insights into the long-term management of neurological manifestations in LSDs^5^.

## Treatment strategies for CNS manifestations

Enzyme replacement therapy (ERT) is a cornerstone in treating lysosomal storage diseases (LSDs), aiming to restore deficient lysosomal enzyme activity and reduce substrate accumulation in affected tissues. LSDs comprise a group of over 50 rare inherited metabolic disorders characterized by deficiencies in lysosomal enzymes, leading to the progressive accumulation of undegraded substrates within lysosomes and subsequent multisystemic manifestations^1^. ERT represents a significant therapeutic advancement, offering a targeted approach to address the underlying enzyme deficiency and alleviate the disease burden in affected individuals. The development of ERT for LSDs stemmed from the understanding of the molecular mechanisms underlying these disorders, particularly the identification of specific lysosomal enzyme deficiencies responsible for substrate accumulation and disease pathology. Early studies in animal models and cell culture systems demonstrated the feasibility and efficacy of enzyme replacement as a potential treatment strategy for LSDs. The success of pioneering studies in the 1990s paved the way for the clinical translation of ERT, leading to the approval of the first ERT product for Gaucher disease in 1991^1^. ERT involves the administration of recombinant lysosomal enzymes via intravenous infusion or other routes of administration to replace the deficient enzyme activity in affected individuals. The exogenously administered enzymes target lysosomes within cells, facilitating the degradation of accumulated substrates and restoring normal lysosomal function. The dosing regimen, frequency of administration, and route of delivery vary depending on the specific LSD, the recombinant enzyme product, and the individual patient’s clinical status^2^. ERT has been successfully implemented in the treatment of several LSDs, including Gaucher disease, Fabry disease, Pompe disease, and mucopolysaccharidoses (MPS), among others. Clinical trials and real-world experience have demonstrated the efficacy of ERT in improving clinical outcomes, reducing disease burden, and enhancing the quality of life for affected individuals. Key therapeutic benefits of ERT include the reduction of hepatosplenomegaly, skeletal abnormalities, hematological abnormalities, and neurological manifestations associated with LSDs^3^. Gaucher disease serves as a paradigmatic example of the success of ERT in LSDs. Gaucher disease is caused by mutations in the GBA gene, leading to deficient activity of the lysosomal enzyme glucocerebrosidase (GCase) and the accumulation of glucocerebroside within lysosomes. ERT with recombinant glucocerebrosidase has been shown to reduce substrate accumulation, improve hematological parameters, and alleviate symptoms such as hepatosplenomegaly and bone pain in affected individuals. Long-term studies have demonstrated ERT’s sustained efficacy and safety in Gaucher disease, highlighting its role as a standard of care in disease management^4^. Similarly, ERT has revolutionized the treatment landscape for other LSDs, including Fabry disease, Pompe disease, and MPS. Fabry disease is characterized by deficient activity of the lysosomal enzyme alpha-galactosidase A (α-Gal A) and globotriaosylceramide (Gb3) accumulation within lysosomes. ERT with recombinant α-Gal A has been shown to reduce Gb3 accumulation, improve renal function, and alleviate symptoms such as neuropathic pain and cardiac hypertrophy in affected individuals^30^. Pompe disease is caused by mutations in the GAA gene, leading to deficient activity of the lysosomal enzyme acid alpha-glucosidase (GAA) and glycogen accumulation within lysosomes. ERT with recombinant GAA has been shown to reduce glycogen accumulation, improve motor function, and prolong survival in affected individuals. Early initiation of ERT in infantile-onset Pompe disease has been associated with better clinical outcomes and improved long-term prognosis, highlighting the importance of timely intervention in disease management^5^. MPS comprises a group of disorders characterized by deficient activity of lysosomal enzymes involved in the degradation of glycosaminoglycans (GAGs), leading to the accumulation of GAGs within lysosomes and subsequent multisystemic manifestations. ERT with recombinant enzymes such as idursulfase (for MPS II) and elosulfase alfa (for MPS IVA) has been shown to reduce GAG accumulation, improve skeletal abnormalities, and attenuate respiratory and cardiac manifestations in affected individuals^6^. Despite the significant therapeutic benefits of ERT, several challenges remain in its implementation and optimization in the clinical setting. These challenges include the high cost of treatment, limited accessibility in certain regions, potential immune responses to exogenous enzymes, and variable response rates among individuals. Additionally, ERT may have limitations in addressing CNS involvement in LSDs due to the blood-brain barrier (BBB) restrictiveness, which limits the delivery of recombinant enzymes to the central nervous system^31^. Substrate reduction therapy (SRT) has emerged as a promising therapeutic approach for the treatment of lysosomal storage diseases (LSDs), offering an alternative strategy to enzyme replacement therapy (ERT) by reducing the production of accumulating substrates within affected cells. LSDs are a group of over 50 rare inherited metabolic disorders characterized by deficiencies in lysosomal enzymes, leading to the progressive accumulation of undegraded substrates within lysosomes and subsequent multisystemic manifestations. SRT aims to modulate substrate levels and restore cellular homeostasis by inhibiting the biosynthesis of accumulating substrates, thereby attenuating disease progression and alleviating clinical symptoms in affected individuals^7^.

The development of SRT for LSDs was driven by advances in understanding the molecular mechanisms underlying these disorders, particularly the identification of key enzymes and pathways involved in substrate biosynthesis and metabolism. Early studies in animal models and cell culture systems demonstrated the feasibility and efficacy of substrate reduction as a potential treatment strategy for LSDs. The success of pioneering studies in the 1990s paved the way for the clinical translation of SRT, leading to the development and approval of the first SRT agents for Gaucher disease and other LSDs^1^. SRT involves the administration of small-molecule inhibitors or pharmacological chaperones that target specific enzymes or pathways involved in substrate biosynthesis. By inhibiting the activity of key enzymes or modulating their function, SRT agents reduce the synthesis and accumulation of accumulating substrates within lysosomes, thereby restoring cellular homeostasis and mitigating disease progression. The dosing regimen, frequency of administration, and route of delivery vary depending on the specific LSD, the mechanism of action of the SRT agent, and the individual patient’s clinical status^2^. SRT has been successfully implemented in the treatment of several LSDs, including Gaucher disease, Fabry disease, and Niemann–Pick disease type C (NPC), among others. Clinical trials and real-world experience have demonstrated the efficacy of SRT in reducing substrate levels, improving clinical outcomes, and enhancing the quality of life for affected individuals. Key therapeutic benefits of SRT include the reduction of hepatosplenomegaly, skeletal abnormalities, hematological abnormalities, and neurological manifestations associated with LSDs^3^. Gaucher disease serves as a paradigmatic example of the success of SRT in LSDs. Gaucher disease is caused by mutations in the GBA gene, leading to deficient activity of the lysosomal enzyme glucocerebrosidase (GCase) and the accumulation of glucocerebroside within lysosomes. SRT agents such as imiglucerase, velaglucerase alfa, and taliglucerase alfa act as competitive inhibitors of glucosylceramide synthase, the enzyme responsible for catalyzing the synthesis of glucocerebroside. By reducing the production of glucocerebroside, SRT agents decrease substrate accumulation, alleviate symptoms such as hepatosplenomegaly and bone pain, and improve clinical outcomes in affected individuals^4^. Similarly, SRT has revolutionized the treatment landscape for other LSDs, including Fabry disease and NPC. Fabry disease is caused by mutations in the GLA gene, leading to deficient activity of the lysosomal enzyme alpha-galactosidase A (α-Gal A) and the accumulation of globotriaosylceramide (Gb3) within lysosomes. SRT agents such as migalastat and lucerastat act as pharmacological chaperones that stabilize mutant α-Gal A enzymes and enhance their lysosomal trafficking and activity. By restoring α-Gal A function, SRT agents reduce Gb3 accumulation, improve renal function, and alleviate symptoms such as neuropathic pain and cardiac hypertrophy in affected individuals^30^. NPC is caused by mutations in the NPC1 or NPC2 genes, leading to impaired cholesterol trafficking and the accumulation of cholesterol and glycosphingolipids within lysosomes. SRT agents such as miglustat act as glucosylceramide synthase inhibitors, reducing glycosphingolipids’ production and attenuating substrate accumulation in affected cells. By modulating lipid metabolism, SRT agents decrease lysosomal storage, improve neurological function, and prolong survival in affected individuals^5^. Despite the significant therapeutic benefits of SRT, several challenges remain in its implementation and optimization in the clinical setting. These challenges include the limited efficacy of SRT in addressing advanced disease stages, the potential for off-target effects and adverse reactions, and the need for long-term adherence to treatment regimens. Additionally, SRT may have limitations in addressing CNS involvement in LSDs due to the blood-brain barrier restrictiveness, which limits the delivery of SRT agents to the central nervous system^6^. Gene therapy and emerging treatments represent promising avenues for the treatment of lysosomal storage diseases (LSDs), offering innovative approaches to address the underlying genetic defects and restore normal cellular function in affected individuals. LSDs are a group of over 50 rare inherited metabolic disorders characterized by deficiencies in lysosomal enzymes, leading to the progressive accumulation of undegraded substrates within lysosomes and subsequent multisystemic manifestations^5^. Gene therapy and emerging treatments leverage advances in molecular genetics, gene editing technologies, and drug development strategies to provide targeted interventions for LSDs, with the potential to revolutionize disease management and improve outcomes for affected individuals.

Gene therapy holds great promise for the treatment of LSDs, offering a targeted approach to address the underlying genetic defects and restore normal enzyme function in affected cells. Gene therapy aims to deliver functional copies of the defective gene or augment endogenous enzyme activity through viral or non-viral vectors, thereby correcting the underlying molecular defect and reducing substrate accumulation within lysosomes^31^. Viral vectors such as adeno-associated viruses (AAVs) and lentiviruses are commonly used for gene delivery because they efficiently transduce target cells and mediate long-term gene expression^1^. One of the pioneering successes in LSD gene therapy is the treatment of mucopolysaccharidosis type I (MPS I), a severe LSD caused by mutations in the IDUA gene encoding alpha-L-iduronidase. Clinical trials have demonstrated the safety and efficacy of AAV-based gene therapy in delivering functional IDUA genes to affected individuals, resulting in sustained enzyme expression, reduced glycosaminoglycan accumulation, and improved clinical outcomes. Long-term follow-up studies have shown durable therapeutic effects and stabilization of disease progression in treated patients, highlighting the potential of gene therapy as a transformative treatment for MPS I and other LSDs^2^. Emerging gene editing technologies such as CRISPR-Cas9 offer exciting possibilities for the treatment of LSDs by enabling precise modification of the disease-causing genetic mutations. CRISPR-Cas9 allows for targeted genome editing by introducing double-stranded breaks at specific genomic loci, followed by homology-directed repair or non-homologous end joining to correct or disrupt the mutant allele. In LSDs, CRISPR-Cas9-mediated gene editing can correct disease-causing mutations, restore normal enzyme function, and mitigate substrate accumulation in affected cells^3^. Recent preclinical studies have demonstrated the feasibility and efficacy of CRISPR-Cas9 gene editing for the treatment of LSDs such as Pompe disease, Gaucher disease, and Niemann–Pick disease type C (NPC)^30^. In Pompe disease, CRISPR-Cas9-mediated correction of mutations in the GAA gene encoding acid alpha-glucosidase has been shown to restore enzyme activity, reduce glycogen accumulation, and improve muscle function in mouse models of the disease. Similarly, in Gaucher disease, CRISPR-Cas9-mediated correction of mutations in the GBA gene has been shown to restore glucocerebrosidase activity, reduce glucosylceramide accumulation, and alleviate disease phenotypes in cell and animal models^4^. In addition to gene therapy and gene editing, other emerging treatment modalities are being explored for LSDs, including small-molecule inhibitors, pharmacological chaperones, substrate reduction therapies, and cell-based therapies. Small-molecule inhibitors target specific enzymes or pathways involved in substrate biosynthesis or accumulation, thereby reducing substrate levels and attenuating disease progression. Pharmacological chaperones stabilize mutant enzymes, enhance their lysosomal trafficking, and increase their activity, restoring cellular homeostasis and mitigating disease phenotypes^2^. Substrate reduction therapies inhibit the synthesis of accumulating substrates, reducing lysosomal storage and ameliorating disease manifestations. Cell-based therapies involve the transplantation of healthy cells or tissues to replace or supplement defective enzymes, providing a sustainable source of functional enzyme activity in affected individuals^30^. Several small-molecule inhibitors and pharmacological chaperones have been developed and evaluated in preclinical and clinical studies for the treatment of LSDs. Miglustat and eliglustat are small-molecule inhibitors of glucosylceramide synthase that reduce glycosphingolipid accumulation and alleviate symptoms in patients with Gaucher disease. Pharmacological chaperones such as migalastat and lucerastat stabilize mutant alpha-galactosidase A enzymes in Fabry disease, enhancing their lysosomal trafficking and activity and improving clinical outcomes^5^. Substrate reduction therapy has been successfully implemented in the treatment of several LSDs, including Gaucher disease, Fabry disease, and Niemann–Pick disease type C. Miglustat, the first approved substrate reduction therapy for NPC, inhibits glucosylceramide synthase, reducing glycosphingolipid accumulation and ameliorating neurological symptoms in affected individuals. Clinical trials have demonstrated the efficacy of substrate reduction therapy in reducing substrate levels, improving clinical outcomes, and enhancing quality of life for patients with LSDs^6^. Cell-based therapies, including hematopoietic stem cell transplantation (HSCT) and enzyme replacement therapy (ERT) using *ex vivo* gene-modified cells, hold promise for treating LSDs by providing a sustainable source of functional enzyme activity in affected individuals. HSCT involves the transplantation of hematopoietic stem cells from a healthy donor into the recipient, where they differentiate into mature blood cells capable of producing the deficient enzyme. HSCT has been shown to improve clinical outcomes and prolong survival in patients with LSDs such as MPS I, MPS II, and Krabbe disease by providing a source of enzyme-producing cells that can migrate to affected tissues and integrate into host organs^31^. ERT using *ex vivo* gene-modified cells represents a novel approach to enzyme replacement therapy by delivering genetically engineered cells that produce and secrete therapeutic enzymes directly into the circulation. Autologous or allogeneic stem cells are isolated from the patient, genetically modified to express the deficient enzyme, and then reinfused into the patient, where they engraft and produce therapeutic enzyme activity levels. This approach offers the potential for sustained enzyme expression, reduced immunogenicity, and improved efficacy compared to conventional ERT^7^. Recent advancements in cell-based therapies, including the development of induced pluripotent stem cells (iPSCs) and genome editing technologies, have expanded the therapeutic possibilities for LSDs. iPSCs can be generated from patient-derived somatic cells, reprogrammed into a pluripotent state, and differentiated into specific cell types for transplantation. Genome editing technologies such as CRISPR-Cas9 can correct disease-causing mutations in patient-derived iPSCs, enabling the generation of genetically corrected cells for transplantation. These approaches offer the potential for personalized cell-based therapies that address the underlying genetic defects and provide long-term therapeutic benefits for patients with LSDs^8^. Despite the significant progress in gene therapy and emerging treatments for LSDs, several challenges remain in their clinical translation and optimization. These challenges include developing safe and effective delivery systems for gene therapy vectors, optimizing gene editing technologies for precise and efficient genome modification, and mitigating off-target effects and immune responses to gene therapy and cell-based therapies.

Additionally, the high cost of treatment, limited accessibility in certain regions, and regulatory hurdles pose barriers to the widespread adoption and implementation of these novel therapies^9^. Symptomatic management of neurological symptoms in lysosomal storage diseases (LSDs) plays a crucial role in improving the quality of life and functional outcomes for affected individuals. LSDs encompass a diverse group of rare inherited metabolic disorders characterized by deficiencies in lysosomal enzymes. This leads to the progressive accumulation of undegraded substrates within lysosomes and subsequent multisystemic manifestations, including profound neurological involvement. While disease-modifying therapies such as enzyme replacement therapy (ERT) and substrate reduction therapy (SRT) target the underlying pathophysiology of LSDs, symptomatic management focuses on alleviating specific neurological symptoms and improving overall patient well-being. Cognitive impairment and developmental delay are common neurological manifestations in many LSDs, including mucopolysaccharidoses (MPS), Niemann–Pick disease, and Tay–Sachs disease^31^. Symptomatic management strategies for cognitive impairment often involve multidisciplinary approaches, including educational interventions, cognitive rehabilitation, and behavioral therapy. Specialized educational programs tailored to affected individuals’ needs can help optimize cognitive development and academic achievement. Behavioral therapy techniques such as applied behavior analysis (ABA) and social skills training can address challenging behaviors and improve social functioning in affected individuals. Additionally, supportive services such as speech therapy, occupational therapy, and physical therapy can help enhance communication skills, motor function, and activities of daily living^1^. Motor dysfunction and movement disorders are common neurological symptoms in LSDs, such as Pompe disease, Gaucher disease, and Fabry disease. Symptomatic management of motor dysfunction often involves physical therapy, occupational therapy, and assistive devices to improve mobility, strength, and coordination. Physical therapy techniques such as stretching, muscle strengthening, and gait training can help optimize motor function and prevent contractures and joint deformities. Occupational therapy interventions focus on activities of daily living, adaptive equipment, and environmental modifications to enhance independence and quality of life^7^. Assistive devices such as wheelchairs, orthotics, and mobility aids can facilitate mobility and improve functional outcomes for affected individuals^2^. Seizures and epilepsy are significant neurological manifestations in LSDs such as Niemann–Pick disease type C, Krabbe disease, and neuronal ceroid lipofuscinoses. Symptomatic management of seizures often involves antiepileptic medications, seizure monitoring, and seizure precautions to reduce seizure frequency and severity^4^. Antiepileptic medications such as levetiracetam, lamotrigine, and valproate are commonly used to control seizures and prevent seizure-related complications. Seizure monitoring techniques such as electroencephalography (EEG) and ambulatory EEG monitoring can help identify seizure activity and guide treatment decisions. Seizure precautions such as safety measures, seizure diaries, and emergency protocols are essential for minimizing seizure-related risks and optimizing patient safety^3^. Sensory deficits and neuropathic pain are common neurological symptoms in LSDs, such as Fabry disease, mucopolysaccharidoses, and Krabbe disease. Symptomatic management of sensory deficits often involves sensory integration therapy, assistive devices, and pain management strategies to improve sensory function and alleviate neuropathic pain. Sensory integration therapy techniques such as sensory stimulation, proprioceptive input, and vestibular stimulation can help optimize sensory processing and enhance functional abilities. Assistive devices such as hearing aids, visual aids, and adaptive equipment can compensate for sensory deficits and facilitate communication and mobility. Pain management strategies such as analgesic medications, physical therapy, and complementary therapies (e.g. acupuncture and massage) can help alleviate neuropathic pain and improve the quality of life for affected individuals^4^. Psychiatric symptoms and behavioral abnormalities are common neurological manifestations in LSDs, such as Niemann–Pick disease, mucopolysaccharidoses, and Tay–Sachs disease. Symptomatic management of psychiatric symptoms often involves psychotropic medications, psychotherapy, and behavioral interventions to address mood disorders, anxiety, aggression, and other behavioral disturbances. Psychotropic medications such as selective serotonin reuptake inhibitors (SSRIs), atypical antipsychotics, and mood stabilizers can help stabilize mood, reduce anxiety, and manage behavioral symptoms. Psychotherapy techniques such as cognitive-behavioral therapy (CBT), supportive therapy, and family therapy can help individuals cope with emotional challenges, improve social skills, and enhance adaptive functioning. Behavioral interventions such as positive reinforcement, structured routines, and environmental modifications can help manage challenging behaviors and promote positive behavior change^30^.

A multidisciplinary approach to care and support is essential for optimizing outcomes and enhancing the quality of life for individuals with lysosomal storage diseases (LSDs). LSDs are a group of over 50 rare inherited metabolic disorders characterized by deficiencies in lysosomal enzymes, leading to the progressive accumulation of undegraded substrates within lysosomes and subsequent multisystemic manifestations. Given the complex nature of LSDs and the diverse array of symptoms they present, a comprehensive and coordinated approach to care is necessary to address the medical, developmental, psychosocial, and functional needs of affected individuals and their families^2^. The multidisciplinary care team for individuals with LSDs typically includes healthcare professionals from various specialties, including medical genetics, pediatrics, neurology, cardiology, pulmonology, gastroenterology, orthopedics, physical therapy, occupational therapy, speech therapy, psychology, social work, and palliative care. Each member of the care team brings unique expertise and perspectives to the management of LSDs, contributing to holistic care that addresses the diverse needs of affected individuals across the lifespan. Medical genetics specialists play a central role in the diagnosis, genetic counseling, and management of LSDs^30^. They conduct thorough clinical evaluations, genetic testing, and diagnostic studies to establish a definitive diagnosis and assess disease severity and progression. Genetic counselors provide information, support, and guidance to individuals and families regarding the genetic basis of LSDs, inheritance patterns, recurrence risks, and available testing and treatment options^5^. They facilitate informed decision-making and empower individuals to make choices that align with their values and preferences. Pediatricians and specialists in pediatric subspecialties collaborate closely with medical genetics specialists to provide comprehensive medical care for children with LSDs^2^. They monitor growth and development, assess organ function, and manage medical complications associated with LSDs, including respiratory infections, cardiovascular abnormalities, gastrointestinal issues, and musculoskeletal problems. Pediatric subspecialists such as neurologists, cardiologists, pulmonologists, and gastroenterologists provide specialized expertise in managing specific organ system involvement and coordinating multidisciplinary care plans tailored to the individual needs of affected children. Neurologists play a critical role in the evaluation and management of neurological manifestations in LSDs, including cognitive impairment, motor dysfunction, seizures, sensory deficits, and behavioral abnormalities^8^. They conduct comprehensive neurological assessments, perform diagnostic studies such as neuroimaging and electroencephalography (EEG), and prescribe appropriate treatments to optimize neurological function and alleviate symptoms. Neurorehabilitation specialists, including physical therapists, occupational therapists, and speech therapists, provide rehabilitative interventions to improve motor function, mobility, communication, and activities of daily living. Psychologists and psychiatrists are integral members of the multidisciplinary care team for individuals with LSDs, providing psychological support, counseling, and mental health services to address the emotional, behavioral, and psychosocial needs of affected individuals and their families^8^. They assess cognitive and emotional functioning, screen for psychiatric disorders, such as anxiety and depression, and provide evidence-based interventions, including cognitive-behavioral therapy (CBT), supportive therapy, and family therapy. They promote resilience, coping skills, and adaptive functioning in the face of chronic illness and help individuals and families navigate the emotional challenges associated with LSD^2^. Social workers play a vital role in connecting individuals and families affected by LSDs with community resources, support services, and financial assistance programs. They provide advocacy, case management, and psychosocial support to address practical needs, such as healthcare access, insurance coverage, housing, transportation, and education. They collaborate with other care team members to develop comprehensive care plans that promote continuity of care, facilitate transitions across healthcare settings, and optimize coordination of services. Palliative care specialists provide holistic care and support to individuals with LSDs and their families, focusing on symptom management, quality of life, and advanced care planning^5^. They address physical, emotional, social, and spiritual needs throughout the disease trajectory, offering pain management, symptom control, psychosocial support, and end-of-life care. They engage in shared decision-making, advance care planning discussions, and goals of care conversations to ensure that care aligns with the values, preferences, and priorities of affected individuals and their families^1^.

## Challenges and future directions

Challenges and future directions in the treatment of lysosomal storage diseases (LSDs) encompass a multifaceted landscape characterized by the interplay of scientific, clinical, ethical, and societal considerations^15^. LSDs are a group of over 50 rare inherited metabolic disorders characterized by deficiencies in lysosomal enzymes, leading to the progressive accumulation of undegraded substrates within lysosomes and subsequent multisystemic manifestations. While significant progress has been made in understanding the pathophysiology of LSDs and developing therapeutic interventions, several challenges persist, and ongoing research efforts are needed to address these challenges and advance the field^16^. Limitations of current treatment approaches represent a significant challenge in the management of LSDs. Despite the availability of enzyme replacement therapy (ERT), substrate reduction therapy (SRT), and other emerging treatments, these interventions have several limitations, including limited efficacy in addressing advanced disease stages, variable response rates among individuals, and challenges related to treatment access, adherence, and cost-effectiveness^17^.

Additionally, current treatments may not adequately address CNS involvement in LSDs due to the blood-brain barrier restrictiveness, which limits the delivery of therapeutic agents to the central nervous system. Furthermore, the long-term safety and durability of treatment effects remain areas of concern, particularly in lifelong therapy for chronic progressive diseases^18^. Research advancements and potential breakthroughs offer promise for addressing the limitations of current treatment approaches and improving outcomes for individuals with LSDs^5^. Recent scientific discoveries in molecular genetics, gene editing technologies, and drug development have led to innovative therapeutic strategies that target underlying genetic defects, restore normal cellular function, and mitigate disease progression. Gene therapy, gene editing, and small-molecule inhibitors represent promising avenues for the development of disease-modifying treatments that address the root cause of LSDs and provide long-term therapeutic benefits^19^.

Moreover, advances in biomarker identification, disease modeling, and personalized medicine approaches can improve diagnostic accuracy, predict disease progression, and optimize treatment selection for individual patients based on their unique genetic profiles and clinical characteristics^20^. The importance of early intervention and personalized medicine underscores the critical need for timely diagnosis, comprehensive assessment, and individualized treatment planning in the management of LSDs. Early intervention can help mitigate disease progression, preserve organ function, and improve long-term outcomes for affected individuals by initiating treatment before irreversible damage occurs. Newborn screening programs expanded genetic testing panels and enhanced diagnostic algorithms have facilitated earlier identification of LSDs, allowing for prompt initiation of therapy and proactive management of disease complications^21^. Personalized medicine approaches integrating genetic testing, biomarker analysis, and disease modeling can inform treatment decisions, tailor interventions to each patient’s needs, and optimize therapeutic outcomes while minimizing risks and adverse effects. Ethical considerations in the treatment of LSDs raise complex and nuanced issues related to equity, access, autonomy, and informed consent. The high cost of treatment, limited availability of orphan drugs, and disparities in healthcare access pose barriers to equitable care for individuals with LSDs, particularly in underserved populations and resource-limited settings^22,23^. Addressing these challenges requires collaboration among stakeholders, including healthcare providers, policymakers, industry partners, patient advocacy organizations, and the broader community. Additionally, ethical dilemmas may arise in the context of emerging technologies such as gene therapy and gene editing, including concerns about safety, efficacy, equity, and unintended consequences^24^. Robust ethical frameworks, regulatory oversight, and transparent communication are essential for navigating these complex issues and ensuring that the benefits of therapeutic innovation are maximized while risks are minimized^1,25^.

## Limitations

One significant limitation of this review is the variability in the scope of the literature covered. The review aimed to include a broad range of LSDs and their neurological manifestations; however, the selection of studies was constrained by the availability of high-quality research on each disease^8,9^. Some LSDs, particularly rarer or less studied ones, may not have been represented comprehensively due to limited data. Consequently, the review may not fully capture the complete spectrum of neurological manifestations across all LSDs. The focus on more well-documented diseases such as Gaucher disease, Fabry disease, and Niemann–Pick disease might overshadow less prevalent conditions, potentially leading to an incomplete picture of the neurological impact of LSDs^10^.

Another limitation pertains to the methodological variability among the studies included in the review. The review encompasses research from diverse sources, including clinical trials, observational studies, and case reports^11^. This heterogeneity in study design and methodology can introduce data variability and affect the findings’ overall consistency. Differences in diagnostic criteria, assessment tools, and outcome measures across studies can make it challenging to draw uniform conclusions^27^. The review attempted to address this issue by evaluating the quality of the studies and integrating findings from multiple sources, but some methodological consistency still needs to be addressed^28^.

Potential publication bias is another concern in the review. Studies with positive or significant findings are more likely to be published, while research with negative or inconclusive results may be underrepresented^12^. This bias can skew the overall perception of the effectiveness of interventions and the prevalence of neurological manifestations. Although the review included a broad range of studies, including those with varying outcomes, it is difficult to eliminate the influence of publication bias on the conclusions drawn^13^. The review also faces limitations related to the evolving nature of the field. Advances in diagnostics, therapeutic approaches, and understanding of LSDs are continually emerging^29^. While the review incorporates recent developments, there must always be a lag between the publication of new research and its integration into review articles. This temporal gap means that some of the latest advancements or findings might not be included, potentially affecting the currency of the review’s content^14^.

Additionally, the review’s focus on English-language literature may limit its comprehensiveness. Research published in other languages might provide valuable insights into the neurological manifestations of LSDs that were not included due to language barriers^15^. This limitation could affect the completeness of the review and potentially omit relevant studies from non-English-speaking regions where unique findings might exist. The review’s reliance on the quality and availability of data from included studies also presents limitations. For instance, some studies may have small sample sizes or limited follow-up periods, which can affect the robustness and generalizability of their findings^10^. The review attempted to account for these issues by assessing study quality and synthesizing data cautiously, but limitations in individual studies inevitably impact the overall conclusions.

Furthermore, the review does not extensively address the impact of genetic and environmental factors on the neurological manifestations of LSDs^16^. While the review covers genetic mutations and molecular mechanisms, the interaction between genetic predispositions and environmental influences on disease progression and neurological outcomes has yet to be explored. Future research could benefit from a more detailed examination of these factors to provide a more nuanced understanding of their role in disease manifestation^17^. The review also acknowledges the complexity of managing neurological manifestations of LSDs, including challenges related to diagnostic accuracy and treatment efficacy^18^. The effectiveness of various therapeutic approaches, such as enzyme replacement therapy and gene therapy, varies depending on the specific LSD and individual patient factors. While the review discusses these treatments, it may need to fully capture the nuances and limitations of their application in different clinical scenarios^19^. The variability in patient responses and the evolving nature of therapeutic strategies are important considerations that require ongoing evaluation and research. Lastly, the review’s emphasis on summarizing existing research may need to fully address areas where evidence is lacking or further investigation is needed. Identifying literature gaps and highlighting future research areas is crucial for advancing the field^20^. Although the review provides a broad overview, specific research questions and areas of uncertainty that require further exploration may need to be more exhaustively covered.

## Summary

The neurological manifestations of lysosomal storage diseases (LSDs), particularly their involvement in the central nervous system (CNS), present complex challenges that significantly impact the quality of life and functional outcomes for affected individuals. The diverse array of symptoms, including cognitive impairment, motor dysfunction, seizures, sensory deficits, and psychiatric symptoms, underscores the multifaceted nature of LSDs and the need for comprehensive, multidisciplinary care approaches. Despite the advances in understanding the pathophysiology of LSDs and the development of therapeutic interventions, including enzyme replacement therapy, substrate reduction therapy, and emerging treatments such as gene therapy and gene editing, several challenges persist. These challenges include limitations in treatment efficacy, accessibility, affordability, ethical considerations related to equitable access, informed consent, and emerging technologies. Moving forward, continued research efforts are needed to address these challenges and advance the field of LSDs. This includes further exploring disease mechanisms, identifying novel therapeutic targets, optimizing treatment strategies, and implementing early intervention and personalized medicine approaches. Collaborative efforts among healthcare professionals, researchers, policymakers, industry partners, and patient advocacy organizations are essential for driving progress, improving outcomes, and enhancing the lives of individuals affected by LSDs.

## Ethical approval

This study received approval from the Institutional Review Board. All participants provided informed consent, and confidentiality and ethical guidelines were strictly followed throughout the research.

## Consent

Informed consent was not required for this article due to the use of publicly available information and data, which was obtained and analyzed in an aggregated and de-identified manner. The study did not involve any direct interaction or intervention with human subjects, and the research findings were based solely on existing public knowledge and data sources. Therefore, no personal information or individual participation was involved, eliminating the need for informed consent.

## Source of funding

The author declared no financial relationships at present or within the previous three years with any organizations that might have an interest in the submitted work.

## Author contribution

C.E.: conceptualization, visualization, supervision, oversight and leadership, and writing of the original draft.

## Conflicts of interest disclosure

The views and opinions expressed in this paper are solely those of the authors and do not necessarily reflect any institution or organization’s official policy or position. The authors declare no conflicts of interest or financial disclosures related to this research.

## Guarantor

Chukwuka Elendu, e-mail: elenduchukwuka@yahoo.com

## Data availability statement

This published article and its supplementary information files include all data generated or analyzed during this study.

## Provenance and peer review

Commissioned and externally peer-reviewed.
